# Contrast Enhancement for Portal Imaging in Nanoparticle-Enhanced Radiotherapy: A Monte Carlo Phantom Evaluation Using Flattening-Filter-Free Photon Beams

**DOI:** 10.3390/nano9070920

**Published:** 2019-06-26

**Authors:** Aniza Abdulle, James C. L. Chow

**Affiliations:** 1 Department of Physics, Ryerson University, Toronto, ON M5B 2K3, Canada; 2 Department of Radiation Oncology, University of Toronto, Toronto, ON M5T 1P5, Canada; 3 Radiation Medicine Program, Princess Margaret Cancer Centre, University Health Network, Toronto, ON M5G 1X6, Canada

**Keywords:** megavoltage portal imaging, Monte Carlo simulation, nanoparticle, flattening-filter-free photon beam

## Abstract

Our team evaluated contrast enhancement for portal imaging using Monte Carlo simulation in nanoparticle-enhanced radiotherapy. Dependencies of percentage contrast enhancement on flattening-filter (FF) and flattening-filter-free (FFF) photon beams were determined by varying the nanoparticle material (gold, platinum, iodine, silver, iron oxide), nanoparticle concentration (3–40 mg/mL) and photon beam energy (6 and 10 MV). Phase-space files and energy spectra of the 6 MV FF, 6 MV FFF, 10 MV FF and 10 MV FFF photon beams were generated based on a Varian TrueBeam linear accelerator. We found that gold and platinum nanoparticles (NP) produced the highest contrast enhancement for portal imaging, compared to other NP with lower atomic numbers. The maximum percentage contrast enhancements for the gold and platinum NP were 18.9% and 18.5% with a concentration equal to 40 mg/mL. The contrast enhancement was also found to increase with the nanoparticle concentration. The maximum rate of increase of contrast enhancement for the gold NP was equal to 0.29%/mg/mL. Using the 6 MV photon beams, the maximum contrast enhancements for the gold NP were 79% (FF) and 78% (FFF) higher than those using the 10 MV beams. For the FFF beams, the maximum contrast enhancements for the gold NP were 53.6% (6 MV) and 53.8% (10 MV) higher than those using the FF beams. It is concluded that contrast enhancement for portal imaging can be increased when a higher atomic number of NP, higher nanoparticle concentration, lower photon beam energy and no flattening filter of photon beam are used in nanoparticle-enhanced radiotherapy.

## 1. Introduction

External beam radiotherapy is a comprehensive term that describes the use of high-energy ionizing radiation to treat cancer. In radiation dose delivery, the beam is conformed to the shape of the tumour using a series of different collimators, and the dimensions as well as location of the tumour are determined by portal images in the treatment unit [1,2]. This imaging is carried out before treatment for beam geometric verification and patient setup so the radiation dose can be delivered to the target with confidence while sparing the surrounding healthy tissues. The portal image is acquired by a megavoltage radiotherapy beam and is detected by a film or electronic portal imaging device [3,4,5].

The effectiveness of nanoparticles (NP) as radiosensitizers, dose enhancers and contrast agents is becoming increasingly recognized [6,7,8]. Nanoparticles are incorporated into radiotherapy and accumulated within the tumour. Gold NP have been found to aggregate due to interactions with glutathione (GSH) present in cytosol. In cancer cells, since the level of GSH is much higher than that in normal cells, more gold NP are found to be accumulated in the tumour compared to the normal tissue. This increases the efficiency of radiotherapy, which is decreased significantly in tumours due to multiple drug resistance and the actions of antiapoptotic protein survivin [9,10]. During treatment when the photon beam irradiates the tumour, NP inside the tumour interact with photons to emit more secondary electrons [11,12,13]. These electrons contribute further damage to cancer cells close to the NP and therefore increase the dose absorbed by the tumour. For example, if an inner shell electron from the *K* shell of an atom is emitted, Auger process occurs and this results in more electrons emitted in the Auger cascades [12]. These liberated electrons cannot travel very far and deposit their energy quickly. The electrons either interact directly with the DNA of the surrounding cancer cells, or they ionize the surrounding water molecules to produce free radicals [14,15]. These ionized water molecules would further damage the DNA in the same way as the liberated electrons.

Nanoparticles are used as a contrast agent, and gold NP are becoming especially popular [16,17,18]. It has long been a struggle to improve the contrast between cancer cells and surrounding healthy tissues to avoid irradiating normal cells, and to increase the dose targeting the tumour with greater confidence in treatment planning and dose delivery. Gold has a high atomic number (*Z* = 79) and, as such, gold NP absorb more photons than soft tissue. In radiotherapy, photons are attenuated mainly through the following interactions: coherent scattering, pair production, Compton scattering and the photoelectric effect [19]. The amount of coherent scattering is insignificant and does not contribute significantly to the total photon interactions. Pair production is only dominant in an energy range higher than 25 MeV, which is generally out of the routine radiotherapy beam range (6–18 MV). The probability of Compton scattering is largely independent of the atomic number of the material, but it is weakly dependent on photon energies. This means that higher-energy photons undergo a greater degree of Compton scattering than low-energy photons. Unfortunately, the result is the relatively uniform incidence of Compton scattering in soft tissue, lung and bone, resulting in a loss of contrast [20]. The most important interaction related to image contrast is the photoelectric effect, and the probability for the occurrence of the photoelectric effect is heavily dependent on the atomic number of the material as well as the energy of the photons. It is well known that the photoelectric effect is dominant in high atomic number media irradiated by low-energy photons in the kilovoltage range [21]. It is the characteristic high atomic number of gold NP that make them effective imaging contrast agents. However, for portal imaging using a megavoltage photon beam, the photoelectric effect may not be so significant compared to computed tomography imaging using a kilovoltage photon beam.

Flattening-filter (FF) photon beams were commonly used in the past for radiotherapy because they produce a relatively flat beam profile at clinically applicable depths. As the flattening filter is thicker in the center, when the photon beam passes through, it results in beam hardening of the photon beam coming out of the collimator. FF photon beams were used mainly because it was easier in manual treatment planning. However, in some current dose delivery techniques such as intensity-modulated radiotherapy and volumetric-modulated arc therapy, flattening filter is not necessary [22,23,24]. Using a flattening-filter-free (FFF) photon beam has some advantages such as a huge increase of beam output and a decrease of linear accelerator head scatter compared to FF beams [25]. With the flattening filter removed from the accelerator head, the FFF beam has more low-energy photons than the FF beam. The increase of low-energy photons in the kilovoltage range for the FFF photon beam has been studied in depth [26,27,28]. Since these low-energy photons are sensitive to the photoelectric effect affecting the contrast in portal imaging, it is worthwhile to investigate the dependency of imaging contrast enhancement on the FFF photon beams with nanoparticle addition in nanoparticle-enhanced radiotherapy.

The aim of this study is to determine the dependencies of contrast enhancement for portal imaging on a photon beam without using a flattening filter, and by varying the beam energy, nanoparticle material and nanoparticle concentration using Monte Carlo simulation.

## 2. Materials and Methods

The contrast enhancement for portal imaging using megavoltage photon beams was determined using Monte Carlo simulation [29]. The Electron Gamma Shower (EGS)nrc-based code was used based on a macroscopic approach [30,31,32]. Phase-space files and photon energy spectra of the 6 and 10 MV photon beams (field size = 10 × 10 cm^2^) with and without applying the flattening filters (i.e., 6 MV FF, 6 MV FFF, 10 MV FF and 10 MV FFF) were generated using the Geant4 and BEAMnrc Monte Carlo codes with verifications carried out and published elsewhere [27,28]. All beam models were based on the Varian TrueBeam linear accelerator (Varian Medical System, Palo Alto, CA). Material data of the NP added to the soft tissue with different concentrations were generated using the EGSnrc-based PEGS4 code (a stand-alone data preprocessing code) [30]. In this study, nanoparticle materials of gold (Au), platinum (Pt), iodine (I), silver (Ag) and iron oxide (Fe_2_O_3_) were used with concentrations equal to 3, 7, 18, 30 and 40 mg/mL. These concentrations were based on small-animal experiments regarding nanoparticle-enhanced radiotherapy [33,34]. A single beam geometry was used to mimic the portal imaging acquisition geometry using the megavoltage radiotherapy beam.

The transmitted intensity, I_t_, was calculated based on the Beer–Lambert Law [35] regarding the equation: I_t_ = I_o_ e^−μx^, using Monte Carlo simulation. I_o_ is the incident intensity and x is the thickness of the material. μ is the mass attenuation coefficient of the material considering various photon interactions, namely, the photoelectric effect, Compton scattering and pair production between the photon and material. The imaging contrast ratio (ICR) was calculated as ICR = [(I_t_ − I_b_)/I_b_]. I_b_ is the transmitted background intensity for material that only contains soft tissue or water [36]. A heterogeneous phantom (30 × 30 × 20 cm^3^) containing slabs of soft tissue and tumour was used to calculate the ICR. The material of the tumour was changed to different nanoparticle materials with different concentrations in nanoparticle-enhanced radiotherapy. Radiotherapy beams of 6 MV FF, 6 MV FFF, 10 MV FF and 10 MV FFF with a field size equal to 10 × 10 cm^2^ were used as the beam sources for portal imaging.

## 3. Results

**Figure 1:**
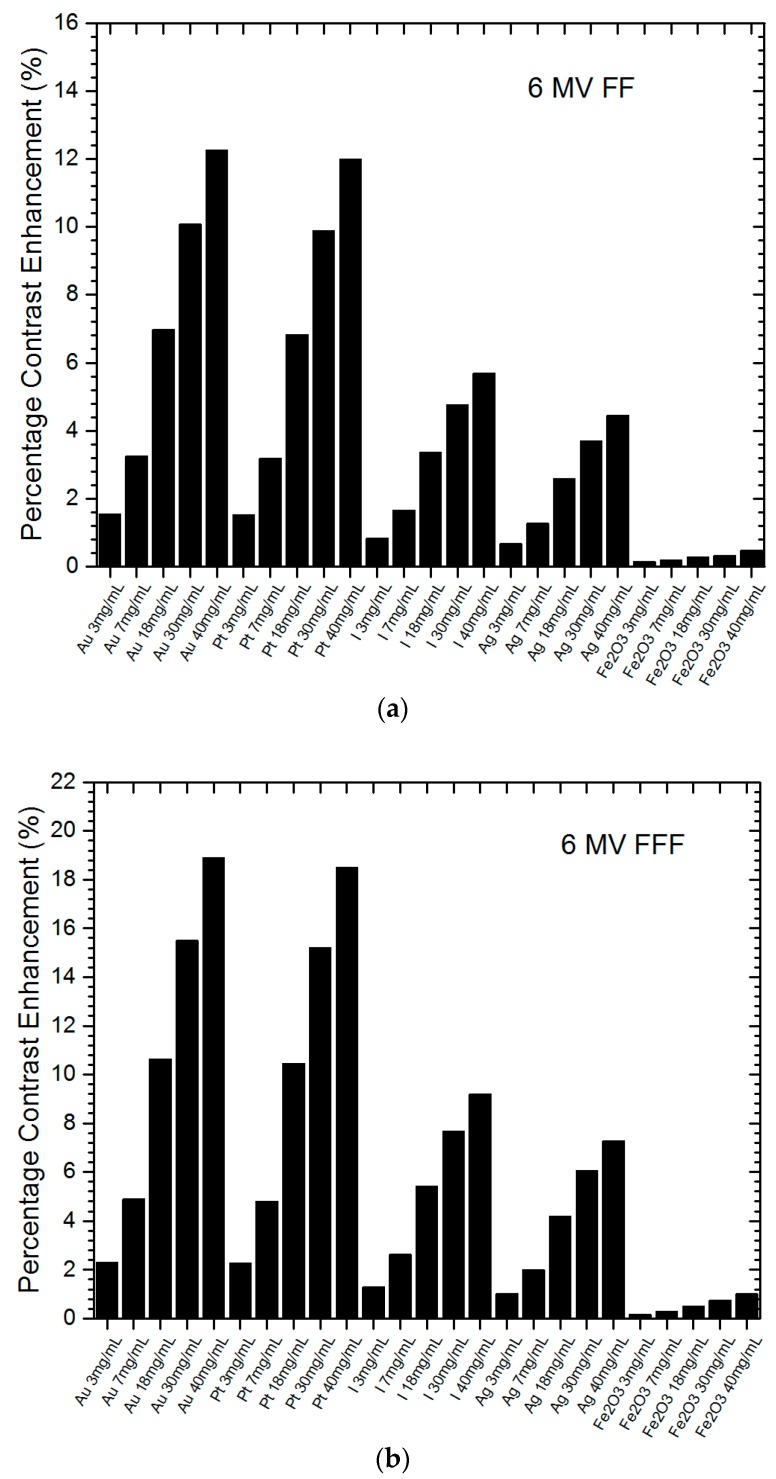
Relationships of percentage contrast enhancement for portal imaging with nanoparticle material and concentration using the 6 MV (**a**) flattening-filter (FF) and (**b**) flattening-filter-free (FFF) photon beams. Gold (Au), platinum (Pt), iodine (I), silver (Ag) and iron oxide (Fe_2_O_3_) nanoparticles (NP) with concentrations equal to 3, 7, 18, 30 and 40 mg/mL were used. The percentage contrast enhancement was calculated as the percentage of increase of imaging contrast ratio when NP were added to the tumour.

**Figure 2:**
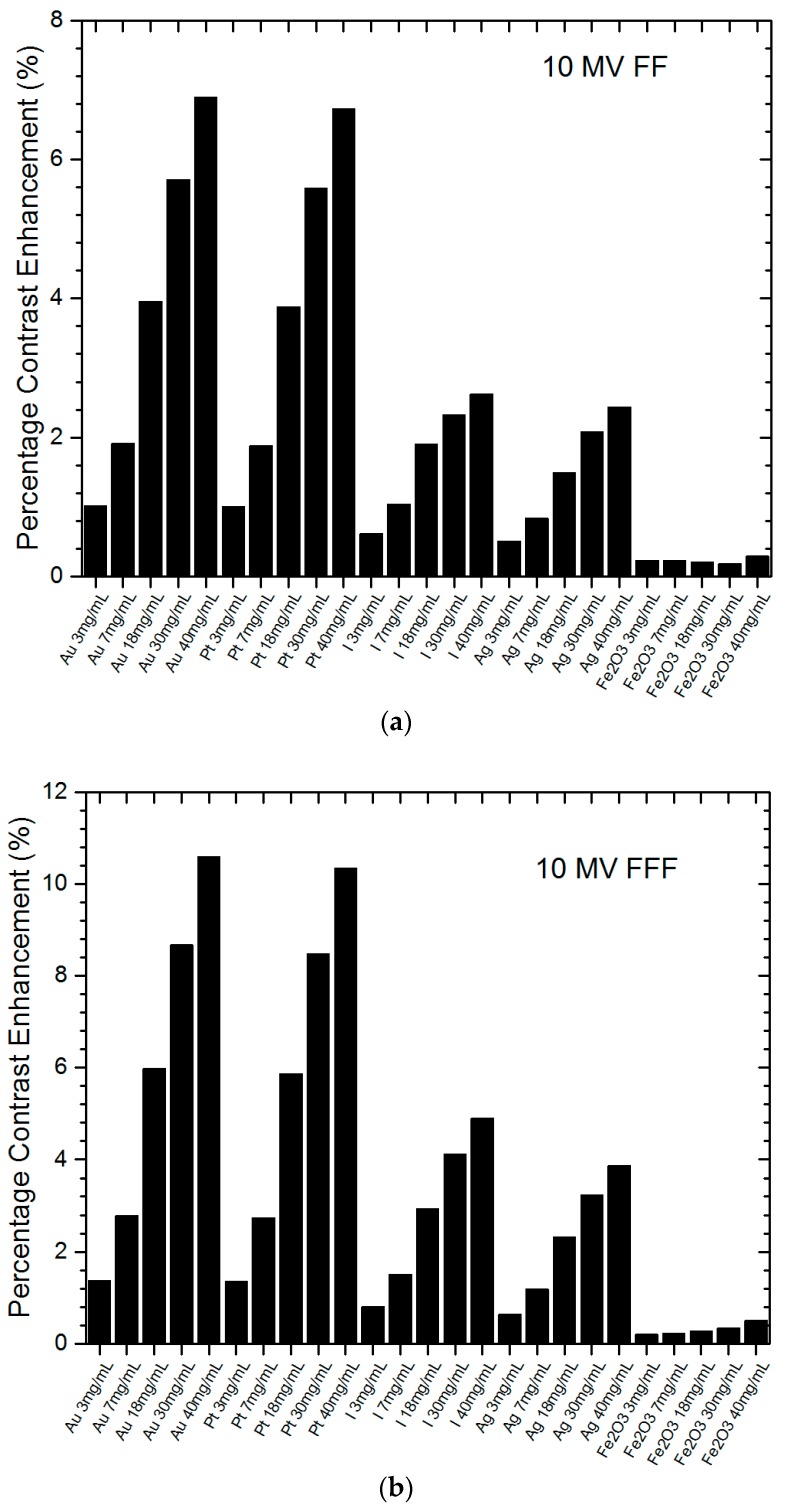
Relationships of percentage contrast enhancement for portal imaging with nanoparticle material and concentration using the 10 MV (**a**) FF and (**b**) FFF photon beams. Gold (Au), platinum (Pt), iodine (I), silver (Ag) and iron oxide (Fe_2_O_3_) NP with concentrations equal to 3, 7, 18, 30 and 40 mg/mL were used. The percentage contrast enhancement was calculated as the percentage of increase of imaging contrast ratio when NP were added to the tumour.

Figure 1a,b shows the relationships of percentage contrast enhancement with the nanoparticle material and concentration using the 6 MV FF and 6 MV FFF photon beams. The percentage contrast enhancement for portal imaging reflected the percentage increase of imaging contrast ratio when NP were added to the tumour, compared to the ratio without adding NP. The nanoparticle material and concentration were plotted together on the *x*-axis of Figure 1 for a better illustration between NP and their corresponding concentration (3–40 mg/mL). It should be noted that zero percentage contrast enhancement means no nanoparticle was added to the tumour, because the contrast ratio does not change with variations of nanoparticle addition. It can be seen that all NP could enhance the portal imaging contrast, and both the gold and platinum NP performed the best among all NP in this study. Figure 2a,b shows similar relationships of percentage contrast enhancement with the nanoparticle material and concentration, using the 10 MV FF and 10 MV FFF photon beams. The maximum imaging contrast enhancement reached about 19% for the gold NP.

## 4. Discussion

### 4.1. Nanoparticle Material

In cancer treatment, heavy-atom contrast agents such as heavy-atom NP can be used to deliver a radiation dose to a tumour while sparing surrounding tissue [37,38]. As this agent enhances the contrast of the tumour in medical imaging, the accuracy of radiation beam targeting is increased. The agent also improves the dose absorption in the tumour and the cancer cell kill. Heavy-atom or high atomic number NP take advantage of a more significant photoelectric effect as the mass attenuation coefficient is proportional to *Z*^3^. However, the photoelectric effect is dominant in the kilovoltage photon beams but not in the megavoltage beams. Therefore, we should not expect the contrast enhancement for portal imaging to be as high as some medical imaging modalities such as computed tomography scanners, which use kilovoltage photon beams as the source [36]. In Figure 1 and Figure 2, it can be seen that high atomic number NP had higher percentage contrast enhancement, especially the gold and platinum NP. The maximum percentage contrast enhancement for the gold NP was 18.9% for the 6 MV FFF photon beam, while for platinum it was 18.5% using the same photon beam and a concentration of 40 mg/mL. The maximum contrast enhancements for the iodine and silver NP were 9.2% and 7.3%, respectively. The iron oxide NP had the lowest percentage contrast enhancement of only 1%. It was found that both the gold and platinum NP produced contrast enhancements about double those of iodine (*Z* = 53) and silver (*Z* = 47), while the iron oxide NP were not so useful in the imaging contrast enhancement. Comparing the toxicity and uptake efficiency to the living cells for the gold, platinum and silver NP, the silver NP were found to be most toxic while the gold NP were non-toxic [39]. Moreover, the gold NP were more easily uptaken by living cells than the platinum and silver [40]. In addition to this, owing to their convenience in fabrication and availability in the market [41], gold NP may become the most popular radiosensitizer in nanoparticle-enhanced radiotherapy.

### 4.2. Nanoparticle Concentration

In Figure 1 and Figure 2, it can be seen that the percentage contrast enhancement increased with an increase of nanoparticle concentration for all nanoparticle materials. This is reasonable, as the amount of photon interaction with NP depends on the nanoparticle concentration. The more NP uptaken by the cancer cell, the more photon-NP interaction and therefore the more absorption of photon fluence in the tumour. This results in an increase of contrast between the nanoparticle-added tumour and the surrounding normal tissue. In Figure 1a, the gold NP increased with a rate of 0.29%/mg/mL, which was very similar to the platinum NP with a rate of 0.28%/mg/mL. These rates were higher than those of the iodine (0.13%/mg/mL) and silver (0.10%/mg/mL) NP. The iron oxide NP had the lowest rate of contrast enhancement, equal to 0.01%/mg/mL. In Figure 1b using the FFF photon beam, the rates of increase of percentage contrast enhancement for the gold, platinum, iodine, silver and iron oxide NP were 0.45, 0.44, 0.21, 0.17, 0.02%/mg/mL, respectively. It was found that both the gold and platinum NP were more sensitive to the change of concentration compared to other NP. In addition, the variation of contrast enhancement was more sensitive when the FFF photon beam was used. When the photon beam energy was increased from 6 to 10 MV, the rates of increase of contrast enhancement for the gold, platinum, iodine, silver and iron oxide NP were 0.16, 0.15, 0.05, 0.05, 0.01%/mg/mL for the 10 MV FF beams (Figure 2a) and 0.25, 0.24, 0.11, 0.09, 0.01%/mg/mL (Figure 2b) for the 10 MV FFF beams. Again, similar trends were found for the variation of imaging contrast enhancement with concentration as seen in the 6 MV photon beams.

### 4.3. Photon Beam Energy

Comparing the 6 and 10 MV photon beams using the flattening filter, as shown in Figure 1a and Figure 2a, it can be seen that the maximum percentage contrast enhancement for the gold NP for the 6 MV beam was higher than that for the 10 MV by about 79%. A similar relationship was found in other NP with higher contrast enhancement in lower photon beam energy (6 MV). This is because the 6 MV photon beam energy spectrum contained more low-energy photons in the kilovoltage range than the 10 MV beam [26]. The extra low-energy photons in the 6 MV beam produced more photoelectric interactions between the photons and NP, resulting in a higher contrast ratio compared to the 10 MV beam. When the flattening filter was removed from the beam, the maximum percentage contrast enhancement of the gold NP for the 6 MV FFF (Figure 1b) beam was 78% higher than the 10 MV FFF (Figure 2b) beam. This proved that nanoparticle addition could benefit the contrast enhancement for portal imaging more when a lower-energy photon beam (6 MV) was used. Moreover, the gold and platinum NP produced the best contrast enhancement for portal imaging with the 6 MV photon beams.

### 4.4. FFF and FF Photon Beams

When the flattening filter is not used in the radiotherapy photon beam, the beam-hardening effect due to the presence of the filter no longer exists. This leads to the FFF photon beam containing more low-energy photons compared to the FF beam [42]. Since those low-energy photons in the radiotherapy beam increase the cross-section of the photoelectric effect, the contrast enhancement is increased in the nanoparticle-added tumour due to its higher compositional atomic number. Comparing Figure 1a,b with the same photon beam energy of 6 MV, it can be seen that the maximum percentage contrast enhancement for the gold NP was 53.6% higher when the flattening filter was not used in the beam. Similar relationships were found in other NP and nanoparticle concentrations. Comparing Figure 2a,b for the 10 MV photon beam, it can be seen that the maximum percentage contrast enhancement for the FFF beam was 53.8% higher than the FF beam for the gold NP. This proves that higher contrast enhancement for portal imaging was obtained using the FFF photon beam in nanoparticle-enhanced radiotherapy.

## 5. Conclusions

Monte Carlo simulation was carried out to predict the dependencies of contrast enhancement for portal imaging on FF and FFF photon beams with various nanoparticle materials and concentrations. It was found that both the gold and platinum NP had the highest contrast enhancement compared to the iodine, silver and iron oxide NP, which have lower atomic numbers. The contrast enhancement was also increased with the nanoparticle concentration, with the rate of increase being the highest for the gold NP, when using the lower beam energy (6 MV) and FFF beam. It is concluded that contrast for portal imaging can be enhanced by nanoparticle additions in nanoparticle-enhanced radiotherapy. High contrast enhancement can be achieved by using high atomic number NP, high nanoparticle concentration, low photon beam energy (6 MV) and FFF photon beams.
